# Impact of Intraoperative Prognostic Factors on Urinary Continence Recovery Following Open and Laparoscopic Radical Prostatectomy

**DOI:** 10.3390/medicina60111824

**Published:** 2024-11-06

**Authors:** Boris M. Kajmakovic, Milos Petrovic, Petar R. Bulat, Uros Bumbasirevic, Bogomir Milojevic, Predrag Nikic, Aleksandar Janicic, Otas Durutovic, Bojan Cegar, Adi Hadzibegovic, Sanja Ratkovic, Zoran M. Dzamic

**Affiliations:** 1 Clinic of Urology, University Clinical Center of Serbia, 11000 Belgrade, Serbia; milospet93@gmail.com (M.P.); bulat.petar@yahoo.com (P.R.B.); urosbu@gmail.com (U.B.); em2bogomir@yahoo.com (B.M.); nicksha@gmail.com (P.N.); aleksandarmjanicic@gmail.com (A.J.); odurutovic@gmail.com (O.D.); bojancegar@gmail.com (B.C.); zorandzamic960@gmail.com (Z.M.D.); 2 Faculty of Medicine, University of Belgrade, 11000 Belgrade, Serbia; 3 Center for Anesthesia and Resuscitation, University Clinical Center of Serbia, 11000 Belgrade, Serbia; a.hadzibegovic@gmail.com

**Keywords:** prostate cancer, radical prostatectomy, laparoscopy, incontinence, bladder neck preservation, posterior reconstruction, anterior suspension, biochemical relapse

## Abstract

*Background and Objectives*: Radical prostatectomy (RP) stands as the predominant instigator of postoperative stress urinary incontinence. Techniques such as the preservation of the neurovascular bundles, bladder neck preservation, and ensuring longer postoperative urethral length have shown positive impacts on continence. The posterior reconstruction is another method that aids in early continence recovery. Anterior suspension as simulator of puboprostatic ligaments is another factor. *Materials and Methods*: This study was conducted in the Clinic of Urology, University Clinical Center of Serbia, between December 2014 and January 2020, employing a prospective, non-randomized comparative design. Data were meticulously gathered from 192 consecutive patients. The process of regaining continence was monitored at intervals of 1, 3, 6, 12, and 24 months after surgery. The main criterion for assessing the level of urinary continence was the number of pads used daily. *Results*: The distribution of overall continence rates in the BNP vs. no-BNP group at 3, 6 and 12 months was 86% vs. 60% (*p* < 0.0001), 89% vs. 67% (*p* < 0.0001), 93% vs. 83% (*p* = 0.022). Continence rates in non-posterior reconstruction group (10%, 22%, 34%, and 54% at 1, 3, 6, and 12 months) were statistically significantly lower (*p* < 0.0001). The patients who underwent urethral suspension exhibited significantly higher rates of overall continence at 1 mo (73% vs. 29%, *p* < 0.0001), 3 mo (85% vs. 53%, *p* < 0.001), 6 mo (89% vs. 62%, *p* < 0.0001), 12 mo (95% vs. 76%, *p* < 0.0001), and 24 mo (93% vs. 81%, *p* = 0.007). Patients who underwent urethral suspension had a four-fold greater likelihood of regaining continence (*p* = 0.015). *Conclusions*: Patients who underwent urethral suspension or BNP or posterior reconstruction had higher continence rates. Only the urethral suspension was found to be a significant prognostic factor of continence recovery.

## 1. Introduction

Prostate cancer (PC) remains one of the most prevalent malignancies in the male population worldwide, afflicting around one million men annually [1]. While radical prostatectomy (RP) has been accepted as a very effective treatment approach, it is not devoid of significant challenges [2]. Male stress urinary incontinence (SUI) primarily arises as an iatrogenic consequence following RP [3]. This condition is characterized by the patient’s report of unintentional urine leakage during physical exertion [4]. RP is the major cause of stress urinary incontinence in the male patient population. As a result, the consequences of RP have a major impact on patients’ quality of life (QoL), often leading to considerable disruptions in their daily activities and routines [5]. In a recent comprehensive examination of functional outcomes, the reported incidence of SUI was 11% three years after RP [6].

Post-prostatectomy urinary incontinence (PPUI) arises due to a combination of factors, prominently involving the injury and deterioration of anterior and posterior pelvic supporting structures and innervation, with consecutive dysfunction of the bladder, urethral sphincter, or both [7,8]. Following RP, urinary leakage is reported by 5–35% of men [9]. Among them, a substantial proportion of 95% have symptoms that are consistent with the clinical profile of stress urinary incontinence (SUI) [8]. This observation remains valid even within the context of advanced robotic-assisted surgical techniques [10]. Moreover, the postoperative phase is frequently associated with a notable decline in the health-related QoL for these patients [11,12].

The incidence of urinary incontinence may vary based on the surgical technique employed. In the context of open radical prostatectomy (ORP), postoperative urinary incontinence rates have been documented to be between 5 and 30% [13,14]. Comparatively, laparoscopic radical prostatectomy (LRP) has shown a rate of 5–21% [9]. Over the last decades, robot-assisted radical prostatectomy (RARP) has emerged as a popular choice, owing to its precision and minimally invasive nature. Despite these advancements, the incontinence rate post-RARP remains a matter of investigation, with studies reporting a broad range of 5–20%. Nevertheless, it should be addressed that these rates are influenced by multiple factors, including surgeon’s expertise, patient selection, and definitions of incontinence across studies. Additionally, according to the longitudinal studies published so far, clinical assessments often reveal improvements in continence over time postoperatively [15,16,17].

Looking back at the past, several surgical techniques have been developed with the aim of improving continence recovery after radical prostatectomy. Techniques such as the preservation of the neurovascular bundles, bladder neck preservation, and ensuring longer postoperative urethral length have all shown positive impact on postoperative urinary continence [18,19]. The posterior reconstruction or “Rocco stitch” is another method that also showed advantage in early continence recovery [20]. Furthermore, preserving the puboprostatic ligaments, which are crucial components of the anterior urethral support system, may be associated with enhanced continence recovery following radical prostatectomy [21]. In RARP, the Retzius-sparing approach has also been noted for its benefits in early urinary continence recovery. Additionally, post-operative pelvic floor muscle training (PFMT) has been endorsed for its role in promoting urinary continence. However, for men with severe postoperative incontinence, the artificial urinary sphincter (AUS) implantation remains a gold standard [22,23,24].

The aim of our study was to evaluate the impact of different intraoperative techniques on urinary continence recovery in patients following ORP and laparoscopic radical prostatectomy (LRP).

## 2. Materials and Methods

This was a prospective, non-randomized, comparative, single-center study conducted in the Clinic of Urology, University Clinical Center of Serbia, Belgrade, between December 2014 and January 2020. The study included 192 patients undergoing ORP (N = 68) or LRP (N = 128) for previously histopathological confirmed PC. Noteworthy, all radical prostatectomies were performed by the same surgeon, with expertise in both laparoscopic and open surgical techniques. The decision on the surgical approach (minimally invasive-laparoscopic or open approach) depended on the personal preference of the patient and in a certain number of cases due to technical limitations due to which laparoscopic radical prostatectomy could not be offered to the patient.

The preoperative diagnostic protocol consisted of measuring prostate-specific antigen (PSA) and performing digitorectal examination (DRE), multidetector-computed tomography (MDCT) of the abdomen and pelvis and bone scintigraphy. Multiparametric magnetic resonance imaging (mpMRI) was not a routine diagnostic tool in this study, and it was performed preoperatively only in selected cases. The preoperative evaluation of the prostate size is conducted using ultrasonography.

Patients who had histologically confirmed prostate cancer (cT3a or less) and opted for surgical treatment were eligible for the current study. This study did not include men with preoperative urinary incontinence, previous medical history of local radiotherapy, and/or urethral surgery. Additionally, men with a history of diseases that might potentially have a significant impact on continence, such as diabetes mellitus, neurological disease, or urethral stricture disease, as well as men with a urinary catheter enabling the evaluation of preoperative continence were also excluded from this study.

### 2.1. Surgical Technique

**Figure 1:**
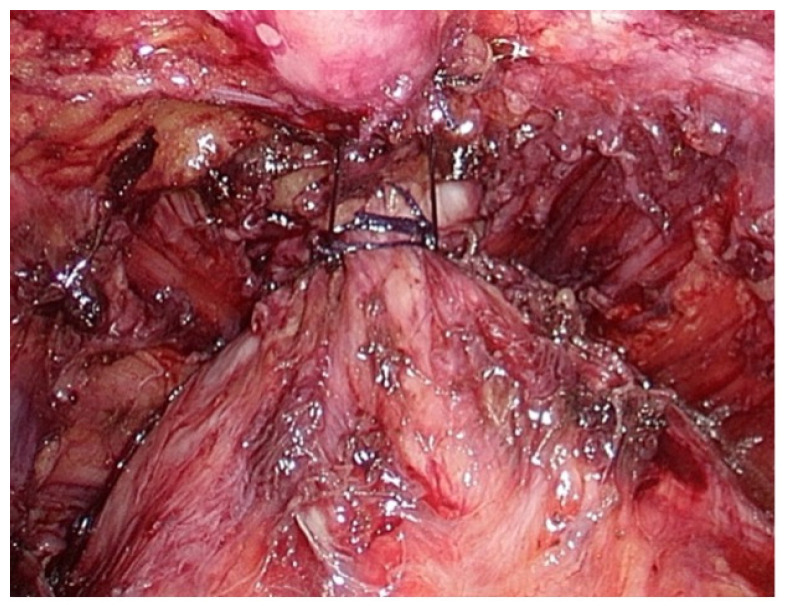
Anterior urethral suspension to the lower portion of the pubic bone periosteum.

Our surgical procedure initiated with dissection of space of Retzius and removal of prostatic anterior fat pad. Following the bilateral incision of the endopelvic fascia and puboprostatic ligaments, as well as the separation of levator ani fibers from the prostatic apex, the Dorsal Vein Complex (DVC) and membranous urethra were subsequently revealed. The ligation of DVC was performed with absorbable suture. Following the completion of DVC suture placement, urethral suspension was performed by anchoring DVC suture to the lower portion of the pubic bone periosteum, Figure 1. Anterior urethral suspension was performed only in patients with an intraoperatively assessed membranous urethra length below 10 mm.

**Figure 2:**
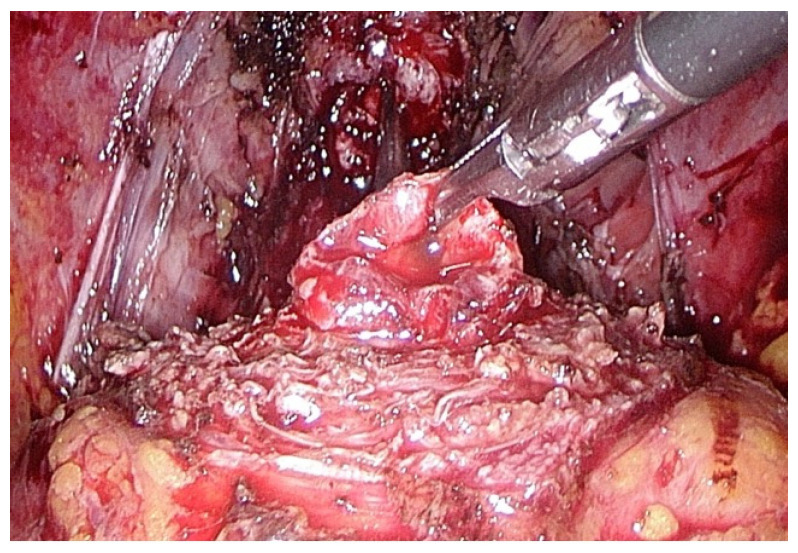
Preservation of the bladder neck.

Following a meticulous identification of the bladder neck, the preservation of the bladder neck was undertaken in patients who did not have a prostatic median lobe, Figure 2. Conversely, the presence of a median lobe required its removal and subsequent reconstruction of the bladder neck.

**Figure 3:**
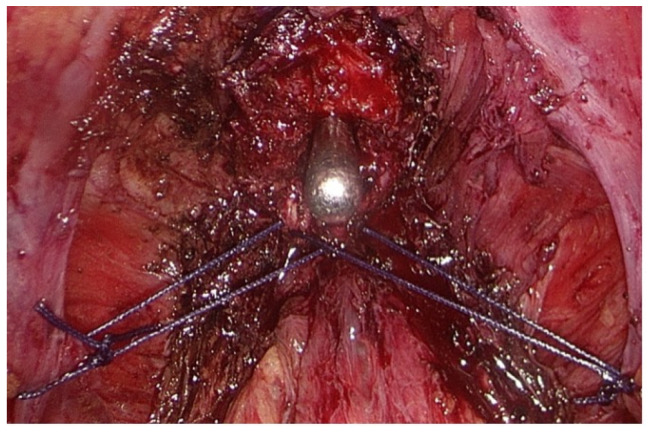
Posterior reconstruction with modified Rocco stitch.

Following the dissection of the seminal vesicles and the incision of Denonvillier’s fascia, the prostatic vascular pedicles were ligated. The sparing of neurovascular bundles was not applied. Following the incision of the prostatic apex and subsequent removal of the prostate, a posterior reconstruction was carried out utilizing a modified Rocco stitch, Figure 3. The technique involved the use of symmetrical stitches that integrated the posterior aspect of the membranous urethra and the cut margins of the endopelvic fascia. Posterior reconstruction was performed solely on patients who had a prostate volume (PV) over 60 mL, as determined by preoperative ultrasonographic examination.

In the laparoscopic approach, the urethro-vesical anastomosis was conducted with a continuous-running 3-0 Monocryl suture, following the method described by Van Velthoven et al. [25]. In the open approach, urethro-vesical anastomosis was performed using six 3-0 polyglactin sutures. A bladder filling-test with saline followed to ensure the anastomosis’s integrity. At the end of the surgery, a closed suction device was used to drain the retropubic space and urethro-vesical anastomosis. The drain was commonly removed the day following the procedure, provided that the drain volume was less than 100 mL.

### 2.2. Postoperative Continence Care and Evaluation

Postoperative continence evaluation was conducted after the surgical procedure. This phase was characterized by proactive patient rehabilitation. To enhance the process of recovery of urinary continence by strengthening the muscles in the pelvic floor, patients were instructed to follow a regimen of Kegel exercises. The urinary catheter was typically removed on the 12th postoperative day, and cystography was not commonly performed prior to catheter removal. The process of regaining continence was closely monitored at intervals of 1, 3, 6, 12, and 24 months after surgery. The main criterion for assessing the level of urinary continence was the number of pads used daily, as self-reported by the patients. In the evaluation of patients’ continence status, a specific criterion was employed based on their responses to the following query: “How many pads did you typically use each day to prevent urinary leakage?” Men who reported using up to two pads per day were categorized as “continent.” Within this group, those who reported zero urinary leakage and no pad usage were subcategorized as “fully continent”. Conversely, men who experienced sporadic urinary leakage necessitating the use of 1–2 pads daily were subcategorized as “socially continent”. Patients who indicated a daily usage of more than two pads were categorized as “incontinent” [20].

### 2.3. Statistical Analysis

The present study involved the comparison of intraoperative parameters with the continence rates at 1, 3, 6, 12, and 24 months postoperatively. Student’s *t*-test was used to compare numerical parameters, while Fischer and Pearson Chi-Square tests were used to assess the discrete variables. To determine potential predictors of postoperative continence recovery, a multivariate logistic regression analysis was used. The statistical analysis was conducted using Statistical Package for Social Sciences 22.0 (SPSS Inc., Chicago, IL, USA), and a value of *p* < 0.05 was considered statistically significant.

### 2.4. Ethical Approval

The study received ethical approval from the Institutional Ethical Board of the Faculty of Medicine, University of Belgrade (approval number 1322/XI-6). All patients who participated in the study provided informed consent.

## 3. Results

In the period from December 2014 and January 2020, a total of 192 patients who met the criteria for inclusion in this study underwent ORP (N = 68, 35%) or LRP (N = 128, 65%) for previously histopathological confirmed PC. Demographic and preoperative characteristics are presented in Table 1, while intraoperative features are summarized in Table 2.

Overall, urethral suspension for pubic bone was performed in 65%, while BNP and posterior reconstruction were conducted in 55% and 34% of patients, respectively. Overall continence rates at 1, 3, 6, 12, and 24 months were 58%, 74%, 79%, 85%, and 88%, respectively. The percentage of fully continent patients (0 pad usage per day) at 1, 3, 6, 12, and 24 months were 19%, 36.5%, 49%, 59%, 66%, and 67%, respectively.

Table 3 displays the results in relation to the surgical approach and continence. No statistically significant difference was seen in the distribution of European Association of Urology (EAU) risk groups for biochemical recurrence between patients selected for LRP and those selected for ORP (*p* = 0.57). Furthermore, no statistically significant association was observed between the surgical approach and continence rates at 1, 3, and 6 months following the surgery. At 12 months post-operation, there was no statistically significant difference in the overall rate of continence between patients who underwent LRP and those who underwent ORP. However, there was a statistically significantly higher proportion of fully continent patients in the LRP group compared to the ORP group (73% vs. 54%, *p* < 0.038). Nevertheless, this statistical significance was not found at the 24th postoperative month.

Table 4 shows the results regarding urethral suspension to the pubic bone and continence. The patients who underwent urethral suspension showed significantly higher rates of overall continence during the entire follow-up period, in comparison to the patients without urethral suspension (*p* value at 1, 3, 6, and 12 month was <0.0001, and 0.007 at 24 month). Similarly, the proportion of patients who achieved full continence (0 pads per day) was shown to be significantly greater among those who underwent urethral suspension during the entire follow-up time (*p* < 0.0001).

A statistically significant association was observed between the preservation of the bladder neck and overall continence rate at 1, 3, 6, 12, and 24 months following the surgery. In a same manner, it was observed that the BNP group showed a significantly higher proportion of patients who were fully continent compared to the no-BNP group during the entire follow-up period (Table 5).

Results regarding posterior reconstruction and continence rate are summarized in Table 6. Patients who underwent posterior reconstruction showed a statistically significantly higher overall continence rate compared to patients who did not undergo posterior reconstruction at 1, 3, 6, and 12 months postoperatively. At the 24-month postoperatively, no statistically significant difference was observed in the overall continence rate between patients who underwent posterior reconstruction and those who did not. The proportion of patients who were fully continent was found to be significantly higher in the posterior reconstruction group compared to the group without posterior reconstruction over the entire 24-month follow-up period.

The multivariate logistic regression analysis was used to identify possible intraoperative prognostic factors of continence at the 12-month following the surgery (Table 7). Variables included in a model were surgical approach, urethral suspension for pubic bone, bladder neck preservation, and posterior reconstruction. Among them, only urethral suspension for pubic bone was found to be statistically significant predictor of continence at the 12-month postoperatively (OR 4.52; 95% confidence interval (CI) 1.42–14.40, *p* = 0.011).

## 4. Discussion

Urinary continence recovery following RP can be influenced by multiple preoperative, intraoperative, and postoperative factors [26]. A 2021 meta-analysis, which included a total of 114 studies, identified patients’ age, membranous urethral length (MUL), prostate volume (PV), and Charlson comorbidity index (CCI) as a significant preoperative predictors of postoperative urinary incontinence (UI) within 3 months following RP. The same prognostic factors, with the exception of CCI, remained predictive for UI after a 12-month period [27]. The increased utilization of prostate MRI has enabled the assessment of preoperative morphometric parameters as possible predictors of postoperative urinary continence recovery. Prostate apex depth ratio (PADR), intravesical prostatic protrusion length (IPPL), and MUL have been identified as significant prognostic MRI parameters in several preoperative predictive models assessing the risk of PPUI [28,29,30]. Therefore, recognizing these preoperative prognostic factors can be of great importance in selecting patients for RP.

Increased understanding of the anatomy of the prostate neurovascular bundles, DVC, puboprostatic ligaments, bladder neck, prostate shape, and urinary sphincter has facilitated in the development of several surgical techniques aimed at improving post-RP urinary continence recovery [9]. In addition to commonly used procedures such as nerve-sparing, posterior reconstruction, bladder neck preservation, puboprostatic ligament preservation, and Retzius-sparing, other authors have investigated alternate techniques. To avoid possible injury of the rhabdosphincter, several studies evaluated the ligation versus non-ligation of DVC. Although a thermal DVC division with selective suture ligation in comparison to suture ligation and subsequent DVC division was associated with shorter operative time and improved early urinary function, 12-month continence rates were equivalent [31,32]. In a study from 2020, Feng et al. reported significantly higher continence rate in patients who underwent DVC suture ligation and subsequent DVC suspension to periosteum compared to those who underwent DVC endoscopic stapling or cut and suture technique [33]. Studies comparing continence rates between continuous and interrupted sutures, or between barbed and monofilament sutures, used for urethro-vesical anastomosis construction, have not shown any significant differences [34,35]. Puboprostatic ligaments (PPLs), in conjunction with the endopelvic fascia, serve as an essential part of anterior urethral support mechanism, enabling stabilization of urethral sphincter and its anchoring to the pubic bone (7). Several small-sized randomized controlled trials (RCTs) have indicated that PPL sparing during RP is linked to improved continence rates compared to non-PPL sparing [36,37,38].

Several non-randomized and RCTs have compared urine function scores and continence rates between RARP and ORP and have consistently found similar outcomes for both techniques [39,40]. Conversely, a recent systematic review concluded that RARP had better outcomes in terms of urine continence compared to LRP [18]. However, the present expenses linked to robot-assisted surgical systems might be excessively costly for a significant number of regions worldwide, hence restricting the utilization of robotic surgery. Consequently, LRP and ORP emerge as feasible alternatives. Currently, there is a scarcity of data in the literature comparing LRP with ORP in terms of postoperative urinary continence. In our prospective, non-randomized study, 68 patients underwent ORP, while the rest (*n* = 128) underwent LRP. Despite the limitations of our study, such as the lack of randomization and the possibility of selection bias, our final statistical analysis showed that there was no significant difference in the distribution of EAU risk groups for biochemical recurrence between patients chosen for LRP and those chosen for ORP. This finding helps to reduce the impact of the aforementioned limitations. In the final analysis, it has been determined that there is no notable difference in continence rates between the surgical approaches at any point during this study, except for a higher proportion of fully continent patients in the LRP group compared to the ORP group at the 12-month postoperatively (73% vs. 54%, *p* < 0.038). However, this statistical significance was not seen at the 24th month following surgery. It is important to note that the surgical approach was not a significant predictor of the continence recovery after 12 months, according to the results of the multivariable logistic regression analysis.

The primary cause for early PPUI has been suggested to be the decrease in the length of the urethral sphincter and the disruption of the posterior median fibrous raphe [20]. To prevent the caudal displacement of the urethral sphincter, a posterior musculofascial plate reconstruction is carried out by attaching the posterior surface of the urethral sphincter to the remaining Denonvillier’s fascia and the posterior wall of the bladder [20]. So far, two randomized clinical studies examining posterior reconstruction in RARP failed to detect any significant enhancement in the restoration of continence, while in the third trial, it was demonstrated that patients only had a faster recovery time to utilizing 1 pad per day [41,42,43]. Nonetheless, findings from two meta-analyses show that, as compared to control groups, posterior reconstruction is linked to higher rates of urinary continence, especially during the early stages of continence recovery [18,44]. Our study found that patients who underwent posterior reconstruction had significantly higher overall continence rates compared to the control group throughout the first 12 months of follow-up. Applying the 0 pad definition, continence was regained in 37%, 66%, 80%, and 92% of patients who underwent posterior reconstruction at 1, 3, 6, and 12 months, respectively. On the other hand, continence rates in non-posterior reconstruction group (10%, 22%, 34%, and 54% at 1, 3, 6, and 12 months, respectively) were statistically significantly lower (*p* < 0.0001). Our study revealed considerably lower percentages of fully continent patients at 1 month and 3 months postoperatively compared to the results of Rocco et al.‘s study (37% vs. 72% and 66% vs. 79%, respectively). Nevertheless, 6 months and 12 months postoperatively, the rates of continence were comparable (80% vs. 86% and 92% vs. 95%, respectively) [20]. In terms of early recovery of continence, our findings align more closely with the results reported by Joshi et al. and Sutherland et al., who observed continence rates of 49% and 63% at the 3-month postoperatively, respectively [41,42]. In our multivariate logistic regression analysis, we found that posterior reconstruction was linked to a higher likelihood of continence recovery. However, this association did not reach statistical significance (OR = 2.64, 95% confidence interval [CI] 0.49–14.198, *p* = 0.259). Finally, in recent years, during RARP, some techniques have been described which incorporate posterior reconstruction and vesicourethral anastomosis suture. The effect of such techniques on fast continence recovery is good, technique is easy to learn and simple to apply [45].

In order to promote continence recovery after RP, several studies have proposed that anterior suspension of the periurethral complex or urethro-vesical anastomosis could improve the stability of the rhabdosphincter [46,47,48,49,50,51]. Nevertheless, the considerable variability in anterior suspension techniques and the absence of a universally accepted approach make comparing the findings of these studies difficult. In a 2001 small-sized study, Sugimura and colleagues described endopelvic anterior urethral stich (EAUS), placed in the levator ani muscle between the cut margins of endopelvic fascia and the anterior urethra, and reported a significantly faster continence recovery in EAUS patient group [46]. Two prospective, single-blind, randomized clinical trials reported earlier continence recovery in patients who underwent urethro-vesical anastomosis suspension to the DVC and the puboprostatic ligaments [47,48]. Finally, several authors evaluated the suspension of anterior aspect of membranous urethra to the lower portion of the pubic bone periosteum. The findings of these studies consistently indicated that patients who underwent anterior suspension had significantly higher continence rates at 3 or 6 months postoperatively compared to the control groups [48,49,50]. To provide additional support to the urethral rhabdosphincter, we performed urethral suspension to the pubic bone in patients with intraoperatively measured membranous urethral length <10 mm. The patients who underwent urethral suspension showed significantly higher rates of overall continence at 1 mo (73% vs. 29%, *p* < 0.0001), 3 mo (85% vs. 53%, *p* < 0.001), 6 mo (89% vs. 62%, *p* < 0.0001), 12 mo (95% vs. 76%, *p* < 0.0001) and 24 mo (93% vs. 81%, *p* = 0.007) in comparison to the patients without urethral suspension. Similarly, the proportion of patients who achieved full continence (0 pads per day) was shown to be significantly greater among those who underwent urethral suspension during the whole follow-up time (*p* < 0.0001). The reported rates of continence following urethral suspension to the pubic bone demonstrate significant variability in the current literature. When implementing 0 pad definition of continence, we observed a much greater rate of continence after 3 months compared to the study conducted by Campeni et al. [48] (52% vs. 32%, respectively). Still, the rates of continence in our study were significantly lower compared to the continence rates reported in Patel et al.‘s study [48] over the entire follow-up period. In our study, when using the criterion of 0 pad usage, the rates of continence in urethral suspension patient group were at 1, 3, 6, and 12 months were 29%, 52%, 66%, and 85%, respectively. Patel and colleagues, on the other hand, reported continence rates of 40%, 93%, 98%, and 98%, respectively, and observed statistically significantly higher continence rates only at 3 months after RALP. The results of our study correspond more closely with the findings of Hurtes and colleagues [50], who reported that patients who underwent urethral suspension achieved continence rates of 26.5%, 42%, and 65% at 1, 3, and 6 months, respectively. Finally, we found that out of all the variables included in the multivariate logistic regression analysis, only urethral suspension showed a statistically significant association with enhanced continence recovery at 12 months following RP. Compared to the patients without urethral suspension, patients who underwent urethral suspension had a four-fold greater likelihood of regaining continence (OR = 4.08, 95% CI:1.312–12.711, *p* = 0.015).

An important component of our study was also the assessment of the possible impact of BNP on the restoration of continence. A meta-analysis from 2016, which included 13 studies, indicated that BNP increases continence rates at 6 months and 12 months postoperatively, without increasing the likelihood of positive surgical margins [51]. Furthermore, BNP may be linked to a reduced risk of bladder neck contracture, ureteral injury, and urethro-vesical anastomosis urine leakage [52]. In our study, patients eligible for BNP were those without intravesical growth of prostatic median lobe. Compared to the no-BNP group, patients who underwent BNP had statistically significantly higher overall and complete continence rates over the entire 24-month follow-up period. The distribution of overall continence rates in the BNP vs. no-BNP group at 3, 6, and 12 months were 86% vs. 60% (*p* < 0.0001), 89% vs. 67% (*p* < 0.0001), and 93% vs. 83% (*p* = 0.022), respectively. The results we observed correspond to the continence rates reported in a 2013 RCT conducted by Nyarangi-Dix et al. In their study, they found that the continence rates for patients with BNP were 87.4%, 88.4%, and 91.6% at 3, 6, and 12 months, respectively. While our study revealed a significant association between BNP and improved continence rates in the univariate analysis, the link was not found in the multivariate logistic regression analysis.

It is important to acknowledge several limitations of our study. Firstly, the lack of randomization could be associated with the presence of confounding effects of different intraoperative techniques on continence recovery. To address this drawback, we used multivariate logistic regression to adjust for potential confounding factors. Secondly, we did not use validated questionnaires for assessment of the continence status following RP. However, to improve data collection, the information regarding pad usage was collected via direct face-to-face patient interviews in an outpatient department. In the conditions of our health system, it was not possible to adequately measure the amount of urine lost during one day by measuring the weight of incontinence pads. For this reason, we can refer to this argument as a study bias. It is a known fact that the degree of postoperative continence in patients with high-risk prostate cancer after radical prostatectomy is lower compared to patients whose disease was stratified into low and intermediate risk [53]. In this study, we did not investigate postoperative continence in subpopulations of patients according to disease risk.

Notable, strengths of our study include prospective design and long follow-up period. In addition, a single surgeon, skilled in both laparoscopic and open surgical methods, who has completed over 100 cases prior to the beginning of the trial, performed all radical prostatectomies. Furthermore, we assessed the impact of the open vs. laparoscopic approach on the recovery of continence, an area in which there are currently insufficient data in the literature.

## 5. Conclusions

Our univariate analysis revealed that patients who underwent urethral suspension to the pubic bone, bladder neck preservation, or posterior reconstruction had significantly higher continence rates compared to patients who did not undergo these procedures. Nevertheless, according to multivariate analysis, only the urethral suspension to the pubic bone was found to be a significant prognostic factor of continence recovery.

## Figures and Tables

**Table 1:** Demographic and preoperative characteristics are presented.

Characteristic	Total
Age (years), mean ± SD	65.56 ± 5.13
PSA, median (IQR)	11.78 (2.47–31.13)
Prostate volume (mL), median (IQR)	40.94 (15–125)
Clinical stage, *n* (%)	
T1c	18 (9)
T2a	91 (47)
T2b	57 (30)
T2c	19 (10)
T3a	7 (4)
Gleason Score after biopsy, *n* (%)	
6 (3 + 3)	76 (40)
7 (3 + 4)	75 (39)
7 (4 + 3)	31 (16)
8 (4 + 4)	10 (5)
EAU risk classification, *n* (%)	
Low risk	60 (31)
Intermediate risk	108 (56)
High risk	24 (13)

SD, standard deviation; IQR, interquartile range; EAU, European Association of Urology; PSA, prostate specific antigen.

**Table 2:** Intraoperative features.

Characteristic	Total
Surgical approach, *n* (%)	
ORP	68 (35)
LRP	124 (65)
Urethral suspension to the pubic bone, *n* (%)	
Yes	124 (65)
No	68 (35)
Bladder neck preservation, *n* (%)	
Yes	105 (55)
No	87 (45)
Posterior reconstruction, *n* (%)	
Yes	64 (34)
No	125 (66)
Operative time (minutes), mean ± SD	161.45 ± 42.50
Intraoperative bleeding (mL), mean ± SD	154.27 ± 149.65

SD, standard deviation; ORP, open radical prostatectomy; LRP, laparoscopic radical prostatectomy.

**Table 3:** Surgical approach and continence.

Continence Rate, *n* (%)	LRP	ORP	*p*-Value
1-month continence rate	72 (58)	39 (57)	0.924
Fully continent	26 (21)	11 (16)	0.698
Socially continent	46 (37)	28 (41)	
Incontinent	52 (42)	29 (43)	
3-month continence rate	92 (74)	50 (73)	0.920
Fully continent	51 (41)	19 (28)	0.140
Socially continent	41 (33)	31 (46)	
Incontinent	32 (26)	18 (26)	
6-month continence rate	101 (81)	51 (75)	0.292
Fully continent	68 (55)	26 (38)	0.089
Socially continent	33 (27)	25 (37)	
Incontinent	23 (18)	17 (25)	
12-month continence rate	112 (90)	58 (85)	0.295
Fully continent	90 (73)	37 (54)	0.038
Socially continent	22 (18)	21 (31)	
Incontinent	12 (10)	10 (15)	
24-month continence rate	110 (89)	59 (89)	0.886
Fully continent	89 (72)	39 (57)	0.071
Socially continent	21 (17)	21 (31)	
Incontinent	14 (11)	8 (12)	

**Table 4:** Urethral suspension to the pubic bone and continence.

Continence Rate, *n* (%)	Yes	No	*p*-Value
1-month continence rate	91 (73)	20 (29)	<0.0001
Fully continent	36 (29)	1 (1)	<0.0001
Socially continent	55 (44)	19 (28)	
Incontinent	33 (27)	48 (71)	
3-month continence rate	106 (85)	36 (53)	<0.0001
Fully continent	65 (52)	5 (7)	<0.0001
Socially continent	41 (33)	31 (46)	
Incontinent	18 (15)	32 (47)	
6-month continence rate	110 (89)	42 (62)	<0.0001
Fully continent	82 (66)	12 (18)	<0.0001
Socially continent	28 (23)	30 (44)	
Incontinent	14 (11)	26 (38)	
12-month continence rate	118 (95)	52 (76)	<0.0001
Fully continent	105 (85)	22 (32)	<0.0001
Socially continent	13 (10)	30 (44)	
Incontinent	6 (5)	16 (24)	
24-month continence rate	115 (93)	54 (81)	0.007
Fully continent	104 (84)	24 (35)	<0.0001
Socially continent	12 (10)	24 (44)	
Incontinent	8 (6)	14 (21)	

**Table 5:** Bladder neck preservation and continence rate.

Continence Rate, *n* (%)	Yes	No	*p*-Value
1-month continence rate	72 (69)	39 (49)	0.001
Fully continent	30 (29)	7 (8)	<0.0001
Socially continent	42 (40)	32 (37)	
Incontinent	33 (31)	48 (55)	
3-month continence rate	90 (86)	52 (60)	<0.0001
Fully continent	52 (49)	18 (21)	<0.0001
Socially continent	38 (36)	34 (39)	
Incontinent	15 (14)	35 (40)	
6-month continence rate	94 (89)	58 (67)	<0.0001
Fully continent	65 (62)	29 (33.3)	<0.0001
Socially continent	29 (28)	29 (33.3)	
Incontinent	11 (10)	29 (33.3)	
12-month continence rate	98 (93)	72 (83)	0.022
Fully continent	82 (78)	45 (52)	0.001
Socially continent	16 (15)	27 (31)	
Incontinent	7 (7)	15 (17)	
24-month continence rate	97 (94)	72 (89)	0.012
Fully continent	81 (77)	47 (54)	0.003
Socially continent	17 (16)	25 (29)	
Incontinent	7 (7)	15 (17)	

**Table 6:** Posterior reconstruction and continence rate.

Continence Rate, *n* (%)	Yes	No	*p*-Value
1-month continence rate	54 (84)	56 (45)	<0.0001
Fully continent	24 (37)	13 (10)	<0.0001
Socially continent	30 (47)	43 (34)	
Incontinent	10 (16)	69 (55)	
3-month continence rate	60 (94)	80 (64)	<0.0001
Fully continent	42 (66)	27 (22)	<0.0001
Socially continent	18 (28)	53 (42)	
Incontinent	4 (6)	45 (36)	
6-month continence rate	62 (97)	88 (70)	<0.0001
Fully continent	51 (80)	42 (34)	<0.0001
Socially continent	11 (17)	46 (36)	
Incontinent	2 (3)	37 (30)	
12-month continence rate	62 (97)	105 (84)	0.009
Fully continent	59 (92)	67 (54)	<0.0001
Socially continent	3 (5)	38 (30)	
Incontinent	2 (3)	20 (16)	
24-month continence rate	59 (92)	107 (87)	0.286
Fully continent	(90)	69 (55)	<0.0001
Socially continent	2 (3)	39 (31)	
Incontinent	5 (7)	17 (14)	

**Table 7:** Multivariate logistic regression analysis for prognostic factors of continence recovery at 12 months post-operation.

Variables	OR (95% CI)	*p*-Value
Surgical approach	0.75 (0.278–2.044)	0.579
Urethral pubic suspension	4.08 (1.312–12.711)	0.015
Bladder neck preservation	1.91 (0.704–5.197)	0.203
Posterior reconstruction	2.64 (0.49–14.198)	0.259

CI, confidence interval; OR, odds ratio.

## Data Availability

Data available on request from the authors.
